# Dr. Gilbert Knapp Ellis

**Published:** 1917-12

**Authors:** 


**Dr. Gilbert Knapp Ellis,** Niagara 1887, died Sept. 19, of diabetes, aged 60, at his home in South Sodus, (Valois) N. Y.

